# SUMO: A Swiss Army Knife for Eukaryotic Topoisomerases

**DOI:** 10.3389/fmolb.2022.871161

**Published:** 2022-04-06

**Authors:** Yilun Sun, John L. Nitiss, Yves Pommier

**Affiliations:** ^1^ Developmental Therapeutics Branch and Laboratory of Molecular Pharmacology, Center for Cancer Research, National Cancer Institute, NIH, Bethesda, MD, United States; ^2^ Department of Pharmaceutical Sciences, College of Pharmacy, University of Illinois, Rockford, IL, United States

**Keywords:** topoisomerases, DNA repair, SUMO, the ubiquitin–proteasome system, topoisomerase inhibitors

## Abstract

Topoisomerases play crucial roles in DNA metabolism that include replication, transcription, recombination, and chromatin structure by manipulating DNA structures arising in double-stranded DNA. These proteins play key enzymatic roles in a variety of cellular processes and are also likely to play structural roles. Topoisomerases allow topological transformations by introducing transient breaks in DNA by a transesterification reaction between a tyrosine residue of the enzyme and DNA. The cleavage reaction leads to a unique enzyme intermediate that allows cutting DNA while minimizing the potential for damage-induced genetic changes. Nonetheless, topoisomerase-mediated cleavage has the potential for inducing genome instability if the enzyme-mediated DNA resealing is impaired. Regulation of topoisomerase functions is accomplished by post-translational modifications including phosphorylation, polyADP-ribosylation, ubiquitylation, and SUMOylation. These modifications modulate enzyme activity and likely play key roles in determining sites of enzyme action and enzyme stability. Topoisomerase-mediated DNA cleavage and rejoining are affected by a variety of conditions including the action of small molecules, topoisomerase mutations, and DNA structural forms which permit the conversion of the short-lived cleavage intermediate to persistent topoisomerase DNA–protein crosslink (TOP-DPC). Recognition and processing of TOP-DPCs utilizes many of the same post-translational modifications that regulate enzyme activity. This review focuses on SUMOylation of topoisomerases, which has been demonstrated to be a key modification of both type I and type II topoisomerases. Special emphasis is placed on recent studies that indicate how SUMOylation regulates topoisomerase function in unperturbed cells and the unique roles that SUMOylation plays in repairing damage arising from topoisomerase malfunction.

## Introduction

### DNA Topoisomerases

**FIGURE 1:**
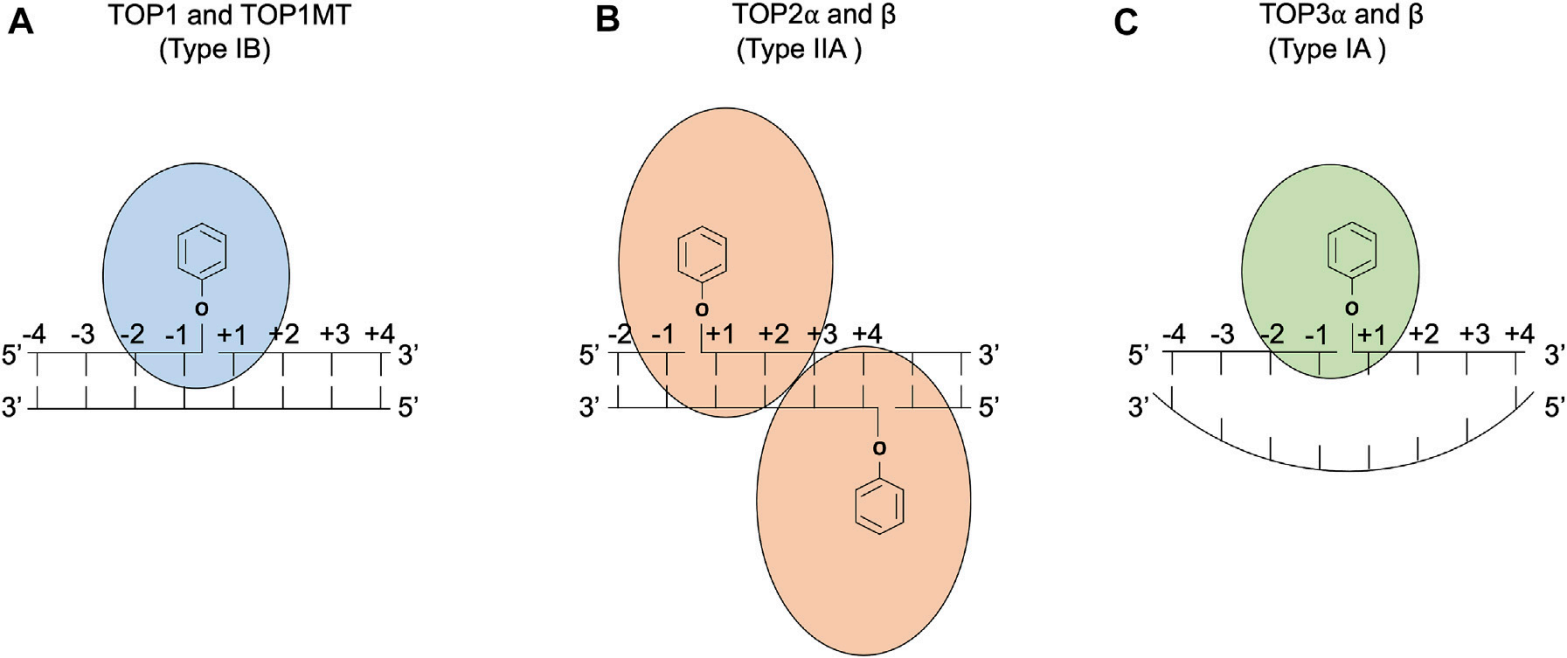
DNA cleavage reactions of topoisomerases. **(A)** Human topoisomerase I and mitochondrial topoisomerase I (TOP1 and TOP1MT) are type IB topoisomerases that form single-stranded breaks (SSBs) by attacking the 3′ phosphate group of the DNA backbone using their active tyrosine residues. **(B)** Human topoisomerases IIα and β (TOP2α and β) are type IIA topoisomerases that form two 5′ covalent linkages between the active tyrosine residues and the 5′ phosphate one on each strand and generate a 4 base-stagger. **(C)** Human topoisomerases IIIα and β (TOP3α and β) are type IA topoisomerases that cleave in single-stranded DNA regions of negatively supercoiled DNA by attacking the 5′ phosphate group with their active tyrosine residues. TOP3β also possesses RNA cleavage activity (Ahmad et al., 2016).

Topoisomerases are ubiquitous enzymes that regulate the topology of DNA during replication, transcription, chromosome condensation, segregation, and other nucleic acid transactions (Chen et al., 2013; Pommier et al., 2016; McKie et al., 2021; Pommier et al., 2022). Topological changes in DNA are accomplished by type I topoisomerases, which act by generation of single-strand breaks, and type II enzymes, which accomplish topological transformations *via* double-strand breaks. There is a wealth of information concerning the biochemical properties of these enzymes, and recent reviews include mechanisms of type 1A enzymes that use a strand passage mechanism (Dekker et al., 2002; Dasgupta et al., 2020), type 1B topoisomerases (Champoux, 2001; Pommier et al., 2016; Seol and Neuman, 2016), and type II enzymes (Vos et al., 2011; Chen et al., 2013; Bush et al., 2015; Hauk and Berger, 2016). The DNA cleavage mechanisms of topoisomerases are summarized in Figure 1. All organisms encode both type 1 and type II topoisomerases, and eukaryotes use type 1A, type 1B, and type II topoisomerases. Human cells have six distinct topoisomerases TOP1, mitochondrial TOP1 (TOP1MT), TOP2α, TOP2β, TOP3α, and TOP3β (Champoux, 2001; Vos et al., 2011; Pommier et al., 2016; Pommier et al., 2022).

Human topoisomerases participate in a broad spectrum of cellular functions. Some of the functions require more than one topoisomerase to cooperate, while the others uniquely require specific topoisomerases. For example, both TOP1 and TOP2α are important for replication, and all the nuclear topoisomerases participate in transcription regulation. By contrast, there are also very specific roles that are unique to specific topoisomerases. For example, TOP1MT does not play roles in nuclear functions (Baechler et al., 2019), and TOP3β uniquely can act on RNA and DNA (Ahmad et al., 2017). Of particular interest is the action of TOP2α. This protein functions in a variety of processes including replication and transcription and also plays specific roles in meiosis (Zhang et al., 2014). Because only type II topoisomerases can catalyze catenation and decatenation of intact double-stranded DNA (Kreuzer and Cozzarelli, 1980; Sundin and Varshavsky, 1981), these enzymes are uniquely required to separate replicated chromosomes that have maintained catenation (Baxter, 2015). A variety of studies specifically showed that TOP2α but not TOP2β is the enzyme that plays this unique role (Linka et al., 2007; McClendon et al., 2008; Gonzalez et al., 2011). TOP2α also plays unique roles in both chromosome structure and chromosome condensation (Maeshima and Laemmli, 2003; Shintomi and Hirano, 2018; Paulson et al., 2021). The utilization of specific topoisomerases in various biological processes remains an area of active investigation, and post-translational modifications are likely to be critical for the recruitment and enzyme activity of specific required enzymes. The potential roles of SUMOylation in these processes are discussed in *SUMOylation in Regulation of Topoisomerases*.

### Topoisomerase-Mediated DNA Damage

The induction of DNA damage by topoisomerases arises from the intermediates of the topoisomerase reaction (Figure 1). While the enzyme normally catalyzes DNA rejoining after performing topological changes, a variety of conditions can prevent the rejoining. These resulting lesions, which consist of a topoisomerase covalently bound to DNA via their normal phosphotyrosyl intermediates, are referred to here as TOP-DPCs (topoisomerase DNA–protein crosslinks). The best studied examples of TOP-DPC induction are small molecules that are anti-cancer topoisomerase-targeting drugs including camptothecins for TOP1 and etoposide and doxorubicin for TOP2. These drugs prevent DNA rejoining by binding to the enzyme–DNA interface and result in their respective TOP-DPCs (Pommier, 2006; Nitiss, 2009b). There are a variety of other conditions that can lead to accumulation of TOP-DPCs. One prominent example is the presence of DNA damage such as oxidative lesions, abasic sites and breaks in DNA, or DNA damage caused by carcinogens. Eukaryotic topoisomerase I appears to be particularly prone to trapping by DNA damage, and abasic sites also efficiently trap TOP2 *in vitro* (reviewed by Nitiss et al. (2019)). In addition, mutations in topoisomerases can impair the enzyme’s ability to carry out re-ligation (Levin et al., 1993; Stantial et al., 2020; Boot et al., 2022).

The diverse ways in which topoisomerases can lead to DNA damage, including a variety of spontaneous mechanisms, suggest that cells have an equally diverse set of pathways for dealing with the damage. Repair of topoisomerase-induced damage has been the subject of many recent reviews (Sun et al., 2020b; Riccio et al., 2020; Zagnoli-Vieira and Caldecott, 2020). In brief, the logic of repair pathways includes detection of a topoisomerase that is trapped (as opposed to an enzyme that is proceeding through the catalytic cycle), removal of the bulk covalently bound protein by proteolysis, removal of the remaining portion of the protein by nucleolytic enzymes such as Tdp1 or non-specific nucleases such as Mre11, and repair of strand breaks by break repair pathways. This logic is not always followed; nucleolytic proteins may act without prior proteolysis (Schellenberg et al., 2017). These mechanisms provide many opportunities for SUMO participation and are discussed in *SUMOylation in Repair of Topoisomerase-Mediated DNA Damage*.

### Overview of the SUMO System

Small ubiquitin-like modifiers (SUMOs) are a class of ubiquitin-like proteins (UBLs) that modify cellular proteins via a three-tiered enzymatic cascade akin to ubiquitin modification (ubiquitylation). The SUMO proteins include a compact globular β-grasp fold and a carboxyl-terminal glycine residue whose pattern of conjugation to substrates also resembles ubiquitylation (Flotho and Melchior, 2013). SUMOylation modulates a broad spectrum of cellular activities ranging from subcellular transport, transcriptional regulation, cell cycle progression, apoptosis, and proteasomal degradation (Geiss-Friedlander and Melchior, 2007; Flotho and Melchior, 2013; Celen and Sahin, 2020).

The mammalian SUMO family includes four members: SUMO-1, SUMO-2, SUMO-3, and SUMO-4, among which SUMO-2 and -3 are nearly identical and share 97% protein sequence identity but only share 42–43% identity with SUMO-1. In general, most SUMOylatable proteins contain a consensus motif Ψ-K-x-D/E in which Ψ is a large hydrophobic residue, K (lysine) is used for SUMO conjugation, and x indicates any amino acid residue, followed by either a (D) aspartic acid or a (E) glutamic acid residue. SUMO-2 and -3 carry internal lysine residues that conform to the consensus motif and hence are capable of polymerizing via isopeptidyl linkage (Flotho and Melchior, 2013; Pichler et al., 2017). In contrast, SUMO-1 lacks the internal consensus motif and is therefore proposed to cap the SUMO-2/3 chain and terminate its elongation (Matic et al., 2008). SUMO-1, SUMO-2, and SUMO-3 are constitutively expressed in all tissues, whereas SUMO-4 expression is restricted to certain tissues (Baczyk et al., 2017) and its functions remain largely unknown.

**FIGURE 2:**
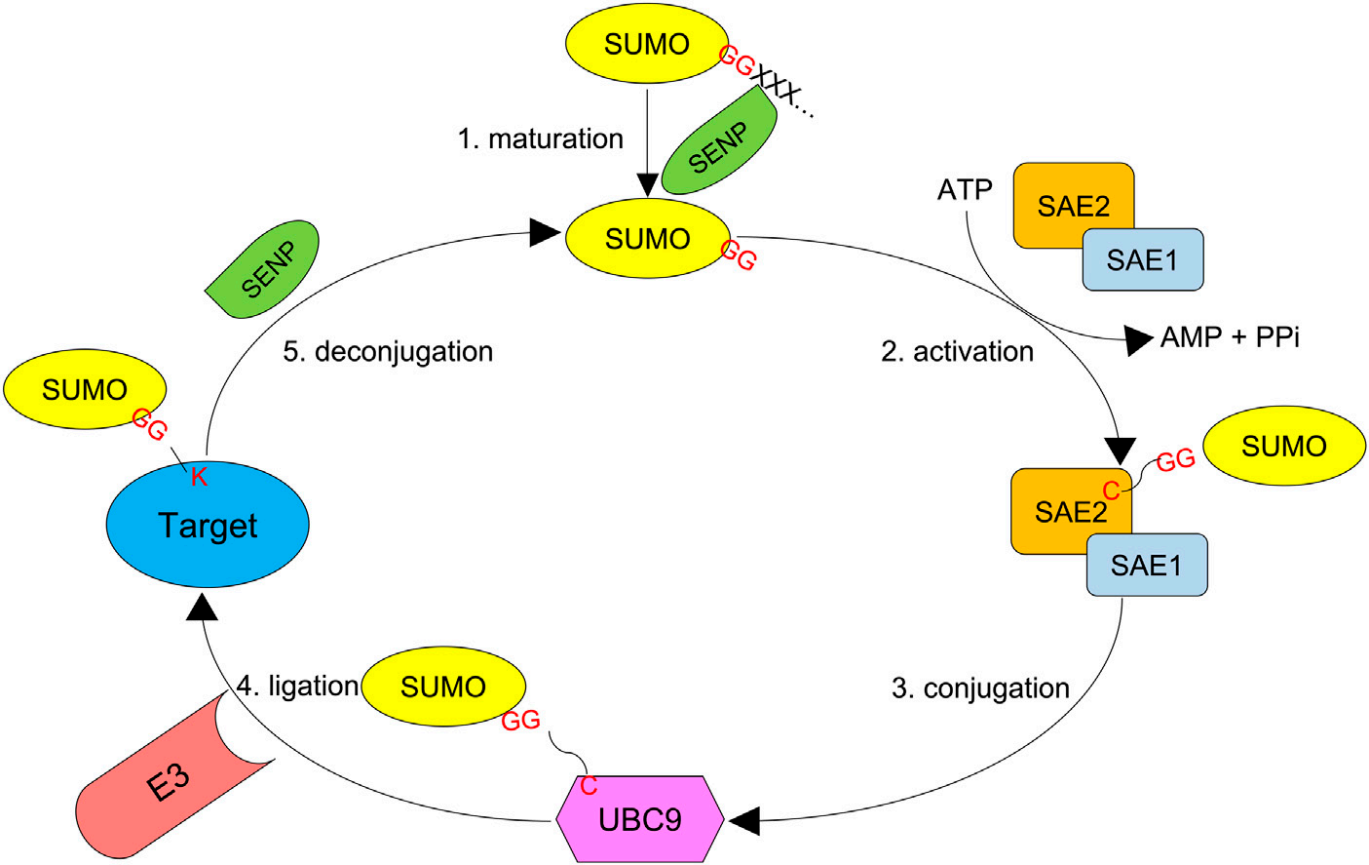
SUMO system. First, the SUMO precursor with additional amino acids at the C-terminus is processed by an SENP protease (Ulp in yeast) to reveal the di-glycine motif. Second, SUMO-activating enzyme subunits 1 and 2 (SAE1 and SAE2), the E1 proteins, dimerize and activate the mature SUMO molecule by forming a thioester bond between their active cysteine residue and the C-terminal glycine of SUMO in an ATP-dependent manner. Third, the activated SUMO is transferred to UBC9, the only SUMO E2 conjugating enzyme. Finally, a SUMO E3 ligase transfers SUMO to the target protein, which forms an isopeptidyl bond between the C-terminal glycine and ε-NH2 of active lysine residue which in most cases resides in a consensus motif Ψ-K-x-D/E. SUMO chains can also be reversed and edited by SUMO proteases.

SUMO acts by modifying target proteins via 1) activation by E1, 2) conjugation by E2, and 3) ligation by E3. SUMO-activating enzyme subunits 1 and 2 (SAE1 and 2) are the two E1 enzymes, and Ubc9 defines the only E2 enzyme. Unlike ubiquitin, whose attachment absolutely requires an E3 ligase, SUMO moieties can be conjugated onto a target protein by the SUMO E2 conjugating enzyme alone as long as the substrate bears a SUMO consensus motif. Although SUMO E3 enzymes (ligases) are dispensable for SUMOylation in many cases, they can recognize specific targets and enhance the efficiency of SUMOylation (Figure 2). For example, SUMO ligases can transfer SUMO proteins to non-consensus lysine residues that are not recognized by Ubc9 (Flotho and Melchior, 2013; Pichler et al., 2017).

The most-studied SUMO E3 ligases are a family of SP-RING–containing proteins termed the Siz and protein inhibitor of activated STAT (PIAS) proteins (Rytinki et al., 2009). The Siz/PIAS family comprises four members including Siz1, Siz2, methyl-methanesulfonate–sensitive protein 21 (Mms21), and molecular zipper protein 3 (Zip3) in the yeast *S. cerevisiae* and five members including PIAS1, PIAS3, PIASxα (PIAS2α), PIASxβ (PIAS2β), and PIASy (PIAS4) in humans. Akin to the RING-type ubiquitin ligases, the Siz/PIAS SUMO ligases bridge substrates and the E2∼SUMO thioester and facilitate SUMO transfer via their SP-RING domains (Hochstrasser, 2001). The nuclear pore complex protein Ran-binding protein 2 (RanBP2) is another type of SUMO E3 ligase. Unlike the Siz/PIAS ligases, RanBP2 bears catalytic domains that interact with E2 and stimulate E2’s SUMOylating activity toward substrates rather than connecting substrates and E2 (Pichler et al., 2004).

SUMOylation can also be deconjugated by a family of cysteine proteases, termed SUMO isopeptidases or SUMO-specific proteases. These SUMO proteases target SUMO-protein conjugates as well as poly-SUMO-2/3 chains by hydrolyzing their isopeptidyl bonds. Also, the SUMO genes encode SUMO precursors that need to expose their C-terminal di-glycine motifs for their adenylation and thioester bond formation by the E1 enzymes. Some of the SUMO proteases serve as processing factors for the C-terminal maturation of the SUMO precursors. The Ulp/SENP (ubiquitin-like protease/sentrin-specific protease) proteases constitute hitherto the largest family of SUMO proteases (Mukhopadhyay and Dasso, 2007; Shin et al., 2012; Nayak and Muller, 2014; Kunz et al., 2018).

SUMO modification is broadly understood to regulate protein localization and alter protein–protein interactions (Flotho and Melchior, 2013). It has been appreciated that SUMO modification can destabilize proteins, and this targeted degradation plays important roles in processes such as DNA repair and homologous recombination (Psakhye et al., 2019). One important class of proteins is the SUMO-targeted ubiquitin ligases (STUbLs). These proteins poly-ubiquitylate extensively SUMOylated proteins (Miteva et al., 2010) to induce their proteasomal degradation. The induction of instability by polySUMOylation is antagonized by SUMO proteases. These processes work together to fine-tune modulation of proteins in space and time.

## SUMOylation in Regulation of Topoisomerases

SUMOylation serves as an important regulatory mechanism for topoisomerase activities and subnuclear localization. In particular, the role of SUMOylation in TOP2 regulation during mitosis has been extensively studied in model systems over the past two decades (Lee and Bachant, 2009; Mukhopadhyay and Dasso, 2017).

**FIGURE 3:**
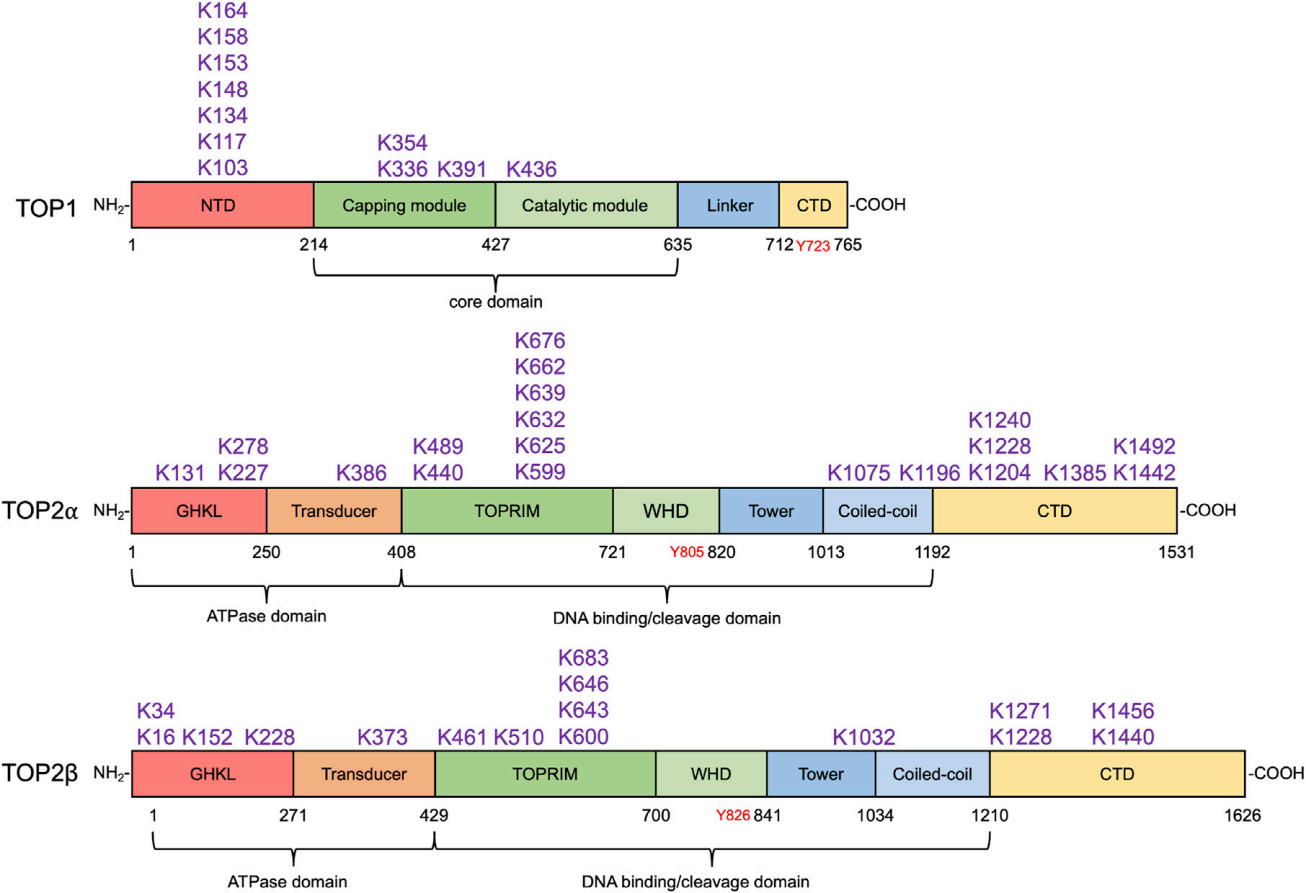
SUMO modification sites in human topoisomerases. SUMOylation sites in human TOP1 and TOP2α and β identified by biochemical and proteomic analyses are shown above their respective domain diagram (Kanagasabai et al., 2009; Hornbeck et al., 2015; Matlock et al., 2015). TOP1 contains the N-terminal domain (NTD), the capping module (CAP), the catalytic module (CAT), the linker, and the C-terminal domain (Takahashi et al., 2022), which are shown in red, darker green, lighter green, light blue, and yellow, respectively. Its catalytic tyrosine residue is shown in red. To date, 11 SUMO modification sites in TOP1 have been identified, most of which locate at the NTD. TOP2α and β are homodimeric enzymes. Each subunit of the homodimer contains an N-terminal ATPase domain bearing the GHKL fold (red) and transducer domain (orange), a central DNA cleavage and ligation domain bearing TOPRIM (topoisomerase-primase) domain (darker green), winged-helix domain (WHD, lighter green), tower (darker blue), and coiled-coil (lighter blue), and a variable C-terminal domain (CTD, yellow) (Nitiss, 2009a). Their tyrosine residues are shown in red. 20 and 16 SUMO modification sites in TOP2α and β have been discovered. SUMO modification sites in human TOP1MT and TOP3α and β have not been identified by any proteomic analyses.

In *S. cerevisiae*, TOP2 is conjugated with Smt3 by Siz1 and Siz2 for localization of TOP2 to the centromeric region, allowing TOP2-dependent decatenation of entangled sister chromatids and faithful chromosome transmission (Takahashi et al., 2006). Genetic studies identified several SUMOylatable lysine residues in yeast TOP2 CTD (K1220, K1246, K1247, K1277, K1278), and mutations in these SUMOylation sites resulted in defects in the cohesion of sister chromatids and in the metaphase-to-anaphase transition (spindle assembly checkpoint) (Bachant et al., 2002). In metazoans, it was originally shown that TOP2α was exclusively modified by SUMO-2/3 but not SUMO-1 on mitotic chromosome in *Xenopus* egg extracts (Azuma et al., 2003). Follow-up studies in the same model system identified PIAS4 as the SUMO ligase that modifies TOP2α with SUMO-2/3 at its lysine 660 (662 in humans, Figure 3) to enrich the protein at the inner centromere of mitotic chromosomes (Azuma et al., 2005; Ryu et al., 2010). Notably, SUMOylation at lysine 660 appeared to suppress TOP2α decatenation activity, raising the possibility that, upon centromeric localization via SUMOylation, TOP2α needs to be deSUMOylated to carry out chromosome decatenation.

Later studies also identified SUMO-2/3 modification at the C-terminal domain (CTD) of TOP2α (Figure 3). Such modification licenses the protein to bind and direct the SIM-containing histone H3 kinase Haspin to centromeres during mitosis (Yoshida et al., 2016). Subsequent phosphorylation of histone tail H3T3 (histone H3 threonine 3) by Haspin recruits the chromosomal passenger complex (CPC) including the kinase Aurora B at kinetochores to ensure proper kinetochore–microtubule attachment and mitosis (Edgerton et al., 2016; Pandey et al., 2020). Cell cycle checkpoint mediator Claspin is another TOP2α CTD–binding partner, and its centromeric enrichment also requires its interaction with TOP2α CTD SUMO-2/3 conjugates *via* the SIMs (Ryu et al., 2015). These findings indicate both lysine 660 (lysine 662 in human TOP2α) and CTD SUMOylation play vital roles for TOP2 during mitotic processes. As opposed to SUMOylating lysine 660, SUMOylating the CTD lysine sites does not impact TOP2α activity. This is likely because lysine 660 resides within the DNA binding/cleavage domain of TOP2 and attachment of the bulky SUMO polymers may affect the conformation of the catalytic core. The CTD SUMOylation, on the contrary, promotes TOP2–partner interactions and TOP2-DPC ubiquitylation (Sun et al., 2020a) without influencing its catalytic activity. Implicit in the findings is the possibility that PIAS4-mediated K660 SUMOylation blocks TOP2α decatenation activity and is not reversed by deSUMOylating enzymes until the chromosomal passenger complex is deployed onto a kinetochore through TOP2α CTD SUMOylation. The CTD SUMOylation also needs to be reversed to release its binding partners for their proper re-localization. This possible mechanism prevents premature sister chromatid segregation and ensures faithful metaphase–anaphase transition.

In agreement with the studies in *Xenopus laevis* egg extracts, it was found in human cells that depletion of PIAS4 exhibited persistently catenated sister chromatids and prolonged metaphase blocks (Diaz-Martinez et al., 2006; Antoniou-Kourounioti et al., 2019). In mouse embryonic fibroblasts (MEFs), proper localization of TOP2α to inner centromeres was found to be driven by SUMO-1 modification by the SUMO E3 ligase RanBP2, a component of nuclear pore complex (nucleoporin) (Dawlaty et al., 2008). Deficiency in RanBP2 results in a chromosome segregation defect including anaphase-bridge formation, leading to aneuploidy and proneness to tumorigenesis. Depletion of PIAS4 in MEFs nonetheless failed to cause any detectable defects in mitosis (Dawlaty et al., 2008). The discrepancy in the role of PIAS4 between model systems requires further interrogation. Given the well-established role of SUMO-1 in facilitating subcellular transport in mammals (Pichler and Melchior, 2002), it can be speculated that SUMO-1 mainly serves as a localization tag for TOP1 and TOP2, whereas SUMO-2/3 modifications play the other regulatory roles such as mediating protein–protein interactions. Very recently, SUMO ligase ZNF451(ZATT) was found to modify TOP2α with SUMO-2/3 on chromatin upon replication stress. SUMOylated TOP2α recruits PICH, PLK1-interacting checkpoint helicase and DNA translocase, to stalled replication forks and resolve topological restraints generated by fork reversal (Tian et al., 2021).

Roles of SUMOylation in TOP1 activity during normal DNA transactions have not been fully exploited. Yet, a study reported that SUMOylation of TOP1 promotes its binding to active RNA polymerase II with hyperphosphorylated CTD (Pol IIo) to attenuate TOP1 activity during transcription (Li et al., 2015). It was shown that the core domain (K391 and K436, Figure 3) of TOP1 is modified with SUMO-1 by the PIAS1–SRSF1 SUMO E3 ligase complex, leading to its co-localization with Pol IIo and the recruitment of RNA splicing factors to the actively transcribed chromatin to reduce R-loop formation. In line with the finding that SUMOylation at lysine 660 of *Xenopus* TOP2α inhibits its decatenation activity, TOP1 lysine K391 and K436 SUMOylation also suppresses its catalytic activity. This finding suggests that deSUMOylation by SUMO proteases is required to reactivate TOP1 upon its successful deployment by SUMOylation.

TOP3 proteins lack a SUMO consensus motif and hence are assumed not to be substrates of SUMOylation. Yet, one study in *S. cerevisiae* reported that the Sgs1–Top3–Rmi1 (STR) helicase–topoisomerase complex is SUMOylated by Mms21, the SUMO E3 ligase component of the SMC5/6 complex, promoting STR to resolve DNA recombination intermediates such as Holliday junctions (Bonner et al., 2016). Sgs1 appears to be the major SUMOylation substrate, and the SUMO modification sites in TOP3 are yet unknown. Whether this SUMO pathway is conserved in mammals warrants further exploration.

## SUMOylation in the Repair of Topoisomerase-Mediated DNA Damage

**FIGURE 4:**
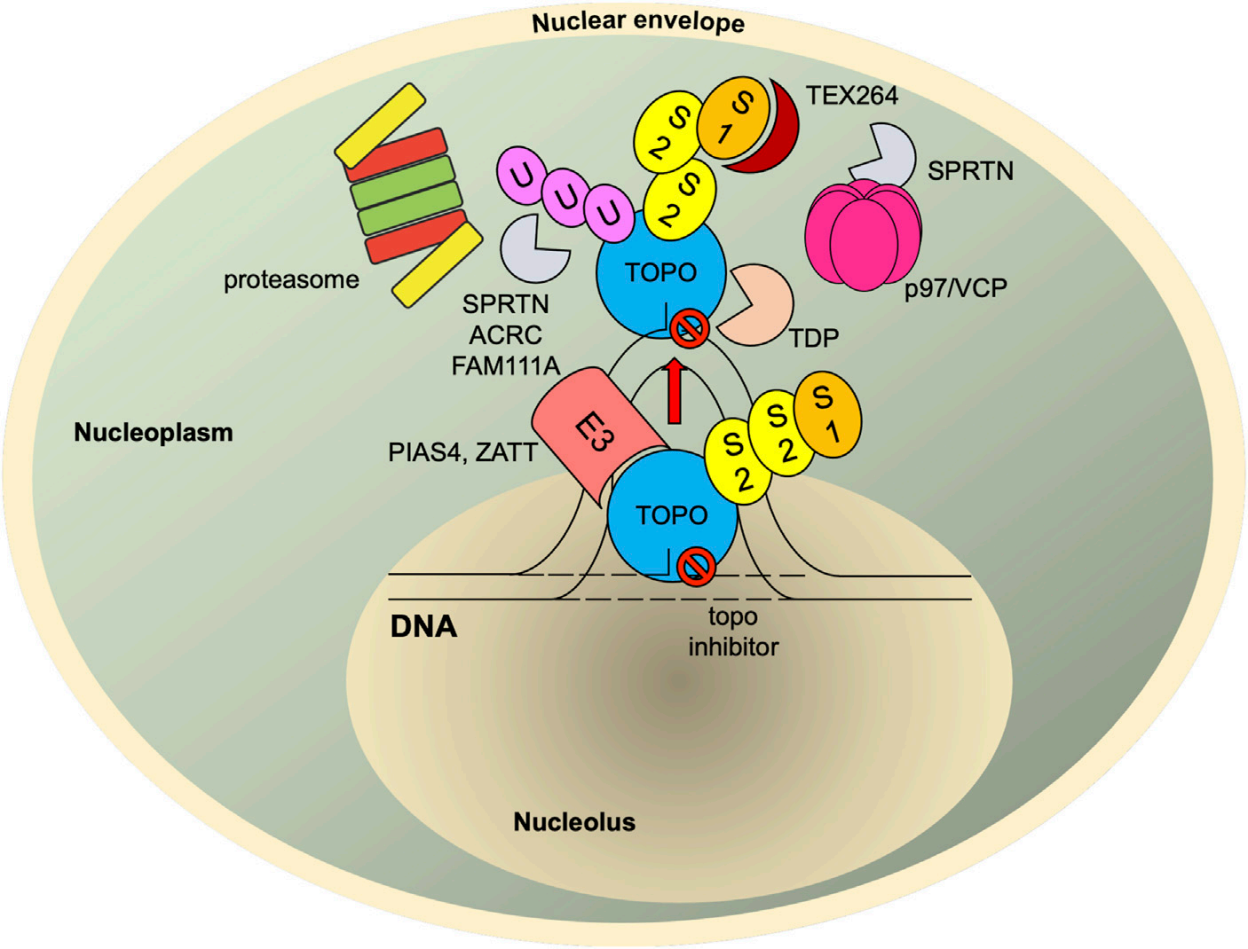
SUMO-mediated TOP-DPC repair. Once TOPcc is trapped and becomes a DPC, it is promptly modified with SUMO-2/3 and then SUMO-1 by PIAS4. The SUMO-1 moieties terminate SUMO-2/3 elongation and relocate TOP1-DPC from nucleolus to nucleoplasm (TOP2 proteins are primarily present in nucleoplasm (Chaly and Brown, 1996)) where RNF4 modifies TOP-DPC with a K48-linked poly-ubiquitin chain. The ubiquitylated TOP-DPC is subsequently targeted by the 26S proteasome for proteolysis. In parallel, the SUMO chain and ubiquitin chain can be recognized and targeted by SPRTN, FAM111A and ACRC. SUMO-1 modification of replication-associated TOP1-DPCs can be read by TEX264, a novel p97 co-factor that subsequently recruits p97/VCP and SPRTN. The p97/VCP hexamer unfolds TOP1-DPC to promote SPRTN-mediated proteolysis and potentially proteasome-mediated proteolysis as well. For TOP2-DPC, its SUMO-2/3 modifications by ZATT also recruit TDP2 to bind and resolve the DPC independently of the proteolysis. S1, SUMO-1; S2, SUMO-2/3; U, ubiquitin.

The SUMO system plays pivotal roles in orchestrating DNA damage responses (reviewed by Chang et al. (2021), Da Costa and Schmidt (2020), and Dhingra and Zhao (2021)). While a detailed discussion of all of these roles is beyond the scope of this article, it is to be expected that SUMO modification might play unique roles in the repair of TOP-DPCs. Indeed, SUMOylation was initially identified as a response to TOP1-DPCs. It was found that SUMO-1 is conjugated to TOP1 in response to camptothecin (CPT), and it occurred almost directly upon drug exposure (Mao et al., 2000b). Later, another study identified K103, K117, and K153 as the three lysine residues at a human TOP1 N-terminus required for its CPT-induced SUMOylation (Horie et al., 2002; Yang et al., 2006) (Figure 4). In *S. cerevisiae*, N-terminal lysine residues K65, K91, and K92 are responsible for TOP1 SUMOylation (Chen et al., 2007). Shortly after the discovery of TOP1 SUMOylation, it was demonstrated that exposure of mammalian cells to teniposide, a TOP2-targeting anti-cancer drug, resulted in SUMO-1 modification of both TOP2α and TOP2β (Mao et al., 2000a). Bisdioxopiperazines such as ICRF-193, a TOP2 inhibitor that traps TOP2 as a circular clamp around DNA, lead to SUMOylation of TOP2 even though it is not covalently bound to DNA (Isik et al., 2003; Xiao et al., 2003).

### TOP-DPC SUMOylation and SUMO-Targeted Ubiquitin Ligases

Ubiquitin-mediated proteasomal degradation of the bulky protein component of TOP-DPCs is a pivotal step in the DPC repair (Desai et al., 1997; Desai et al., 2001; Mao et al., 2001; Desai et al., 2003; Xiao et al., 2003; Lin et al., 2008; Lin et al., 2009; Sun et al., 2020b), and SUMOylation has been established to act as a signal to the ubiquitin–proteasome pathway (UPP) under certain circumstances including DNA damage responses (Uzunova et al., 2007; Galanty et al., 2009; Miteva et al., 2010; Galanty et al., 2012; Yin et al., 2012; Vyas et al., 2013; Chang et al., 2021). The initial experiments in mammalian cells could demonstrate that SUMOylation of TOP-DPCs occurred but were unable to directly demonstrate that the observed SUMOylation was a repair reaction, i.e., SUMOylation led to proteasomal degradation of TOP-DPCs, and that the degradation enhanced cell survival. Early genetic experiments in *S. cerevisiae* demonstrated that reduced UBC9 activity conferred CPT sensitivity in yeast (Jacquiau et al., 2005). A more direct connection between SUMOylation and TOP-DPC repair was obtained in the yeast *S. pombe*. Two SUMO ligases, the SMC5/6 SUMO ligase Nse2 (Mms21 in *S. cerevisiae*) and the PIAS/Siz SUMO ligase Pli1 (Siz1 in *S. cerevisiae*), were found to SUMOylate TOP1-DPCs and recruit the SUMO-targeted ubiquitin ligase (STUbL) (Miteva et al., 2010; Chang et al., 2021) Slx8 to remove the DPCs (Heideker et al., 2011; Steinacher et al., 2013). Mutation in SUMO ligase/STUbL is lethal in combination with other DNA repair defects. Notably, the lethality is suppressed when *Top1* is deleted (Chen et al., 2007; Heideker et al., 2011). Note that TOP1 is not essential for viability in either *S. cerevisiae* or *S. pombe*. These experiments can be simply interpreted as follows: SUMO ligases and STUbL are essential for repairing DNA damage arising from TOP1-DPCs that result from trapping of TOP1 by lesions in the DNA. These experiments highlight the importance of repair pathways for TOP-DPCs under normal growth conditions.

A recent set of experiments demonstrates more directly SUMO modification of topoisomerases leading to proteasomal degradation of both TOP1- and TOP2-DPCs (Sun et al., 2020a). TOP1- and TOP2-DPCs undergo rapid and sequential SUMO-2/3 and SUMO-1 modifications by PIAS4 via its DNA-binding SAP. RNF4, the human ortholog of Slx8 in *S. pombe*, targets the SUMOylated TOP-DPCs binding the SUMO polymers through its SIM (SUMO-interacting motif) domains and ubiquitylates the DPCs for their subsequent degradation by the proteasome (Sun et al., 2020a). This SUMO–ubiquitin pathway also operates in an analogous fashion in *S. cerevisiae* with Siz1 serving as the main SUMO ligase and Slx5–Slx8 ubiquitylating the topoisomerases in a SUMO-dependent manner. Notably, it was found that this SUMO-mediated ubiquitin–proteasome pathway (UPP) was not contingent on either replication or transcription and that inhibition of the pathway did not fully abolish the removal of TOP-DPCs, suggesting the presence of additional UPPs and proteolysis mechanisms recruited by DNA transactions. It has been well established that there are repair pathways for TOP-DPCs that depend on ongoing transcription (Lin et al., 2008) or replication (D'Arpa et al., 1990). While the UPP has been proposed to play a role upon collisions with TOP-DPCs and active DNA transaction machinery such as advancing replication forks or transcription bubbles (Xiao et al., 2003; Lin et al., 2009), the roles of SUMOylation in these detection processes remain to be elucidated.

How does RNF4 recognize TOP-DPCs and thereby trigger its degradation? Given the role of SUMOylation in subnuclear transport of TOP2 during cell division, one possibility is that SUMOylation relocates topoisomerases from chromatin to other nucleoplasmic compartments once topoisomerases reseal DNA, and failure of topoisomerases to release from DNA (persistent TOPcc and trapped closed clamp conformation) can be sensed by PIAS4, which is associated with topoisomerases even in the absence of inhibitors. In consequence, PIAS4 SUMOylates the trapped TOPcc for the UPP-mediated degradation. In agreement with this hypothesis, several earlier observations showed that SUMO-1 modifications of TOP1 induced by camptothecins led to re-localization of the protein (Mo et al., 2002; Rallabhandi et al., 2002; Christensen et al., 2004). These findings suggest that TOP1-DPCs may be directed by SUMOylation to other sites where the lesions may be (Nie et al., 2012) repaired or sequestered (Figure 4).

A recent study reported that Siz2, an analog of Siz1, SUMOylates the vicinity of DPCs (chromatin-bound proteins adjacent to DPCs) to recruit Wss1, a replication-associated metalloprotease carrying the SprT domain, that binds and digests DPCs via its SIMs (Serbyn et al., 2021). Another study in *S. cerevisiae* identified the AAA ATPase Cdc48 (also known as p97 or valosin-containing protein (VCP) in mammals) and its substrate-recruiting adaptor Ufd1/Doa1 as a binding partner of Wss1 (Balakirev et al., 2015). Wss1 was found to form a ternary complex with Cdc48 and Ufd1 to bind and process SUMOylated TOP1-DPCs. The Cdc48 complex is a hexameric unfoldase/segregase that unfolds and segregates protein substrates in an ATP-dependent manner (van den Boom and Meyer, 2018). Ufd1, the adaptor of the Cdc48 complex, specifically recognizes ubiquitylated proteins via its ubiquitin-binding domain (Bodnar et al., 2018). Ufd1 also bears SIMs, making the Cdc48 complex a selective receptor for proteins that are both SUMOylated and ubiquitylated (Nie et al., 2012). It is therefore conjectured that, upon TOP-DPC SUMOylation and ubiquitylation by the Siz1–Slx5/8 pathway, the Cdc48 complex binds the heterologous SUMO–ubiquitin chain and unfolds TOP-DPCs to facilitate their digestion by Wss1. In mammalian cells, SUMOylation was shown to channel DPC repair pathway(s) to SPRTN, the ortholog of Wss1, during DNA replication (Ruggiano et al., 2021). Unlike Wss1, SPRTN lacks SIM but is still able to interact with both ubiquitin and SUMO moieties conjugated to DPCs. This raises the possibility that the PIAS4–RNF4 pathway also recruits SPRTN for TOP-DPC proteolysis and that SPRTN binds the ubiquitin–SUMO hybrid conjugates using its ubiquitin-binding UBZ domain (Kuo et al., 2016; Liu et al., 2021). This model is further supported by a recent study in human cells, which identified TEX264 (testes-expressed 264), a gyrase inhibitory-like protein, as a novel p97 co-factor that recognizes SUMO-1–modified TOP1-DPCs with its putative SIMs and recruits p97 and SPRTN for the repair (Fielden et al., 2020).

ACRC (acidic repeat–containing protein), also known as GCNA (germ cell nuclear antigen-1), is a SPRTN-related protease metalloprotease that repairs DPCs in metazoans in parallel with SPRTN. ACRC is highly expressed in germ cells while maintaining low levels in somatic and most cancer cells. A recent study shows that formaldehyde-induced DPCs are rapidly modified SUMO polymers, which recruit ACRC to the DPC sites *via* its SIMs for proteolysis (Borgermann et al., 2019). ACRC/GCNA was later found by two concurrent studies to co-localize with TOP2 on the mitotic chromosome in *C. elegans* and suppress TOP2-dependent damage during meiosis in *Drosophila* (Bhargava et al., 2020; Dokshin et al., 2020). In human germ cell tumors, ACRC/GCNA removes TOP2-DPCs to prevent replication stress and copy number variation (Bhargava et al., 2020). It is yet to be determined whether SUMOylation is required for ACRC to act against TOP2-DPCs. Emerging evidence also suggests proteases FAM111A (mammal) and Ddi1 (yeast) as parallel pathways for TOP1-DPC repair during DNA synthesis (Kojima et al., 2020; Serbyn et al., 2020). Whether SUMOylation plays a regulatory role for these proteases warrants further investigations.

### SUMOylation and Topoisomerase-Specific Repair Factors

Proteolysis by the proteasome or other proteases cannot entirely remove topoisomerase adducts from DNA, since the protein phosphotyrosyl linkage would not be expected to be a protease substrate. Under most circumstances, proteolysis will leave a short peptide of undetermined length bound to DNA. Therefore, an additional step, complete removal of the peptide, will be needed. All eukaryotes have specialized enzymes for complete removal of the peptides, the tyrosyl phosphodiesterases (TDPs (Zagnoli-Vieira and Caldecott, 2020)) Notably, under many conditions, TDPs require at least partial proteolysis in order to efficiently remove TOP-DPCs (Debethune et al., 2002; Interthal et al., 2005; Gao et al., 2014). Both TDP1 and 2 appear to be modulated by SUMOylation through different mechanisms.

TDP1 was initially identified as an excision nuclease specialized for 3′ phosphotyrosyl adducts including TOP1-DPCs (Yang et al., 1996; Pouliot et al., 1999; Interthal et al., 2005). Subsequent studies demonstrated activity against 5′ phosphotyrosyl adducts, consistent with a role in the repair of both TOP1 and TOP2-DPCs (Nitiss et al., 2006; Murai et al., 2012). TDP1 was found to be modified by all three SUMO isozymes (primarily SUMO-1) at its N-terminal lysine 111 within its consensus motif in mammalian cells (Hudson et al., 2012). TDP1 SUMOylation facilitates its recruitment to TOP1-DPCs and does not appear to affect its cleavage activity (Hudson et al., 2012). The SUMO E3 ligases specific for TDP1 SUMOylation have not been identified, and it is possible that PIAS4 may act on TDP1 to coordinate ubiquitylation and TDP1-dependent hydrolysis for TOP1-DPC repair through SUMOylation.

Human TDP2 has been shown by proteomic studies to carry two non-canonical SUMO modification sites (Hendriks et al., 2014; Matlock et al., 2015), but their impacts on TDP2 remain unknown. A study reported that ZNF451 (ZATT), a zinc-finger–containing SUMO ligase, targets TOP2α- and β-DPCs for SUMO-2/3 modifications, which recruit TDP2 to target the TOP2–DNA junction without proteolysis (Schellenberg et al., 2017). It was shown that TDP2 binds SUMOylated TOP2-DPCs via its split-SIM motifs and releases the intact protein with formation of protein-free DSBs. However, it remains enigmatic when and how the cell employs ZATT to obviate the need for proteolysis. The SUMOylation sites in TOP2 for ZATT are yet to be discovered. Since ZATT-mediated SUMO-2/3 modification is proposed to alter the conformation of TOP2-DPCs and expose its tyrosine–DNA linkage, such modification likely takes place in the DNA binding and cleavage domain, bridging TDP2 and the exposed scissile bond.

Overall, SUMOylation plays key roles in repair of TOP-DPCs. SUMO ligase activity targeting the DPCs clearly contributes to proteolysis by proteasome and non-proteasome proteases. There are several key questions that will likely yield important insights. Given the large number of pathways by which TOP-DPCs can be processed, one of the major questions is pathway choice. It is noteworthy that we understand recognition that is independent of transcription and replication better than we understand pathways that are initiated upon inhibition of replication or transcription. The regulation of modification of topoisomerase-specific repair proteins such as TDPs as well as general repair nucleases such as the MRE11 complex and proteases such as SPRTN will lead to new insights for these questions.

## Perspectives and Concluding Remarks

TOP1 and TOP2 are important substrates for SUMOylation, and the role of SUMOylation for topoisomerases has been extensively explored within the past two decades. It yet remains enigmatic what SUMO is exactly doing for topoisomerases. SUMOylation seems to act as an early responder to covalently trapped TOP1 and TOP2 by priming them for the repair by proteases and nucleases (Sun et al., 2020b). SUMOylation is also required for proper subnuclear localization and protein–protein interaction of free topoisomerases to maintain genome stability (Yoshida and Azuma, 2016). The distinct purposes of SUMOylation are determined by different cellular contexts such as replication, transcription, chromosome segregation, and driven by different E3 ligases, different SUMO isozymes, and different SUMO modification sites in the enzymes (Table 1). For example, the nucleoporin RanBP2 is likely employed to modify free TOP2 with SUMO-1 and localize it to inner centromeres during mitotic phases, whereas the SAP DNA-binding domain-containing E3 ligase PIAS4 deposits SUMO-2/3 to the CTD of centromere-bound TOP2 to promote its interactions with chromosome passenger proteins (Dawlaty et al., 2008; Ryu et al., 2010; Yoshida and Azuma, 2016). During transcription, chromatin-bound TOP1 is modified with SUMO-1 at its core domain by PIAS1 to bind Pol IIo and RNA splicing factors (Li et al., 2015).

**TABLE 1:** SUMO ligases and deSUMOylating enzymes for topoisomerases.

SUMO E3 ligase	Substrate	References
PIAS1 (Siz1 in *S. cerevisiae*)	TOP1	Li et al. (2015)
PIAS4	TOP1-DPC, TOP2α, and TOP2α- and TOPβ-DPC	Azuma et al. (2005); Sun et al. (2020a)
RanBP2	TOP2α	Dawlaty et al. (2008)
ZATT/ZNF451	TOP2α, TOP2α- and TOPβ-DPC	Schellenberg et al. (2017); Tian et al. (2021)
Topors	TOP1	Hammer et al. (2007)
**DeSUMOylating enzyme**		
Ulp2/Smt4 (*S. cerevisiae*)	TOP2	Bachant et al. (2002)
Ulp1? (*S. cerevisiae*)	TOP1	Chen et al. (2007); Serbyn et al. (2021)

Once trapped on chromatin, TOP1- and TOP2-DPCs are first modified with SUMO-2/3 and then SUMO-1 by PIAS4 (with the assistance of PIAS1 (Galanty et al., 2009; Kumar et al., 2017)). TOP1-DPCs are SUMOylated at the NTD, whereas TOP2-DPCs are SUMOylated at the CTD (Sun et al., 2020a). SUMOylation primes both DPCs for their ubiquitylation that is conjugated either to the existing SUMO chains or to lysine residues adjacent to the SUMO chains (Miteva et al., 2010; Kravtsova-Ivantsiv and Ciechanover, 2012), leading to proteolytic degradation by proteasome and other proteases. The reason why the NTD of TOP1 and the CTD of TOP2 are enriched with SUMO modification sites is likely because these two domains are poorly structured and hence inherently susceptible to proteasomal degradation (Berko et al., 2012). Of note, this PIAS4–RNF4 pathway has recently been demonstrated to prompt proteasomal degradation of DNMT1 (DNA methyltransferase 1) DNA–protein crosslinks (Liu et al., 2021). A more recent study shows that the PIAS4–RNF4 pathway recruits the unfoldase VCP/p97 to repair non-covalent PARP1–DNA complexes trapped by PARP inhibitors (Krastev et al., 2022). These findings indicate SUMO modification as a universal repair mechanism for both covalent DNA–protein crosslinks and non-covalent DNA–protein complexes.

SUMO-1 modification of TOP1-DPCs is also implicated to relocate the DPC from nucleolus to nucleoplasm (Rallabhandi et al., 2002). Such subnuclear delocalization is critical, as the proteasome system is absent from nucleolus (Chen et al., 2002). Studies have revealed that nuclear 26S proteasomes are enriched at and tethered to the nuclear pore complex (Enenkel, 2014; Albert et al., 2017). Interestingly, one study in *S. cerevisiae* reported that SUMOylation relocates DSB to the nuclear pore where the UPP degraded SUMOylated proteins in the vicinity of replication-associated DSBs to facilitate DSB repair with the pore proteins (Horigome et al., 2016; Kramarez et al., 2020). Likewise, it can be postulated that SUMO-1 modification of TOP-DPCs directs them to the nuclear envelope for proteasomal degradation and the subsequent repair of the DPC-associated DSB. Similarly, the novel p97 co-factor TEX264 was found to localize to TOP1-DPC–stalled replication forks near the nuclear periphery and repair SUMO-1–modified TOP1-DPCs with p97 and SPRTN (Fielden et al., 2020).

SUMO modification sites for human TOP1MT and TOP3 have not been described in detail although SUMOylation of TOP3 in yeast has been reported (Bonner et al., 2016). Due to the lack of the SUMOylation site–rich NTD of its nuclear equivalent, TOP1MT may not be a substrate of SUMOylation (Zhang et al., 2001). The identification of SUMOylation of TOP1 and TOP2 has certainly been fostered by the availability of selective inhibitors. The lack of potent and specific inhibitors of type 1A topoisomerases renders exploration of SUMOylation of these enzymes more challenging.

While the results presented throughout this review suggest that SUMOylation of TOP1 and TOP2 can be divided into SUMOylation to promote specific biological processes and SUMOylation that is specifically part of repair pathways, the division may not be as clear as this review suggests. The normal biological function of topoisomerases may depend on their degradation (see, for example, Guturi et al. (2016)), and the repair pathways may represent extensions of pathways related to normal processes. As another speculative example of this way of thinking about topoisomerase degradation, TOP3 degradation appears to be important in appropriate resolution of recombination intermediates (Dhingra and Zhao, 2021).

Since topoisomerases are critical substrates for SUMOylation, it is of interest to consider the extent to which SUMOylation defects arise from its failure to properly act on topoisomerases or TOP-DPCs. SUMOylation is an important stress response, and non-genotoxic stresses can alter the SUMOylation of chromatin proteins that include topoisomerases (Bradley et al., 2021). It is clear that SUMO mediates genome stability (Jalal et al., 2017); to what extent does that mediation involve topoisomerases?

Finally, it will be important to identify deSUMOylating enzymes for topoisomerases and TOP-DPCs. For example, what are the SUMO proteases for TOP2α during mitosis? How are the proteases recruited to timely reverse PIAS4-mediated SUMOylation and promote the decatenation activity of TOP2α? Answers to these questions will require meticulous investigations. Since the 26S proteasome lacks deSUMOylating activity and is unable to unfold SUMOylated substrates, close coordination between SUMO proteases and the proteasome will be required (Ciechanover and Stanhill, 2014).

## References

[B1] Ahmad M. Xue Y. Lee S. K. Martindale J. L. Shen W. Li W. (2016). RNA Topoisomerase Is Prevalent in All Domains of Life and Associates with Polyribosomes in Animals. Nucleic Acids Res. 44, 6335–6349. 10.1093/nar/gkw508 27257063 PMC4994864

[B2] Ahmad M. Xu D. Wang W. (2017). Type IA Topoisomerases Can Be "magicians" for Both DNA and RNA in All Domains of Life. RNA Biol. 14, 854–864. 10.1080/15476286.2017.1330741 28534707 PMC5546716

[B3] Albert S. Schaffer M. Beck F. Mosalaganti S. Asano S. Thomas H. F. (2017). Proteasomes Tether to Two Distinct Sites at the Nuclear Pore Complex. Proc. Natl. Acad. Sci. U.S.A. 114, 13726–13731. 10.1073/pnas.1716305114 29229809 PMC5748218

[B4] Antoniou-Kourounioti M. Mimmack M. Porter A. Farr C. (2019). The Impact of the C-Terminal Region on the Interaction of Topoisomerase II Alpha with Mitotic Chromatin. Ijms 20, 1238. 10.3390/ijms20051238 PMC6429393 30871006

[B5] Azuma Y. Arnaoutov A. Dasso M. (2003). SUMO-2/3 Regulates Topoisomerase II in Mitosis. J. Cel. Biol. 163, 477–487. 10.1083/jcb.200304088 PMC2173648 14597774

[B6] Azuma Y. Arnaoutov A. Anan T. Dasso M. (2005). PIASy Mediates SUMO-2 Conjugation of Topoisomerase-II on Mitotic Chromosomes. EMBO J. 24, 2172–2182. 10.1038/sj.emboj.7600700 15933717 PMC1150894

[B7] Bachant J. Alcasabas A. Blat Y. Kleckner N. Elledge S. J. (2002). The SUMO-1 Isopeptidase Smt4 Is Linked to Centromeric Cohesion through SUMO-1 Modification of DNA Topoisomerase II. Mol. Cel. 9, 1169–1182. 10.1016/s1097-2765(02)00543-9 12086615

[B8] Baczyk D. Audette M. C. Drewlo S. Levytska K. Kingdom J. C. (2017). SUMO-4: A Novel Functional Candidate in the Human Placental Protein SUMOylation Machinery. PLoS One 12, e0178056. 10.1371/journal.pone.0178056 28545138 PMC5435238

[B9] Baechler S. A. Dalla Rosa I. Spinazzola A. Pommier Y. (2019). Beyond the Unwinding: Role of TOP1MT in Mitochondrial Translation. Cell Cycle 18, 2377–2384. 10.1080/15384101.2019.1646563 31345095 PMC6739053

[B10] Balakirev M. Y. Mullally J. E. Favier A. Assard N. Sulpice E. Lindsey D. F. (2015). Wss1 Metalloprotease Partners with Cdc48/Doa1 in Processing Genotoxic SUMO Conjugates. Elife 4, e06763. 10.7554/eLife.06763 PMC4559962 26349035

[B11] Baxter J. (2015). "Breaking up Is Hard to Do": The Formation and Resolution of Sister Chromatid Intertwines. J. Mol. Biol. 427, 590–607. 10.1016/j.jmb.2014.08.022 25194916

[B12] Berko D. Tabachnick-Cherny S. Shental-Bechor D. Cascio P. Mioletti S. Levy Y. (2012). The Direction of Protein Entry into the Proteasome Determines the Variety of Products and Depends on the Force Needed to Unfold its Two Termini. Mol. Cel. 48, 601–611. 10.1016/j.molcel.2012.08.029 PMC5603081 23041283

[B13] Bhargava V. Goldstein C. D. Russell L. Xu L. Ahmed M. Li W. (2020). GCNA Preserves Genome Integrity and Fertility across Species. Dev. Cel. 52, 38–52.e10. 10.1016/j.devcel.2019.11.007 PMC6946843 31839537

[B14] Bodnar N. O. Kim K. H. Ji Z. Wales T. E. Svetlov V. Nudler E. (2018). Structure of the Cdc48 ATPase with its Ubiquitin-Binding Cofactor Ufd1-Npl4. Nat. Struct. Mol. Biol. 25, 616–622. 10.1038/s41594-018-0085-x 29967539 PMC6044470

[B15] Bonner J. N. Choi K. Xue X. Torres N. P. Szakal B. Wei L. (2016). Smc5/6 Mediated Sumoylation of the Sgs1-Top3-Rmi1 Complex Promotes Removal of Recombination Intermediates. Cel Rep. 16, 368–378. 10.1016/j.celrep.2016.06.015 PMC5051638 27373152

[B16] Boot A. Liu M. Stantial N. Shah V. Yu W. Nitiss K. C. (2022). Recurrent Mutations in Topoisomerase IIα Cause a Previously Undescribed Mutator Phenotype in Human Cancers. Proc. Natl. Acad. Sci. U.S.A. 119, e2114024119. 10.1073/pnas.2114024119 35058360 PMC8795545

[B17] Borgermann N. Ackermann L. Schwertman P. Hendriks I. A. Thijssen K. Liu J. C. (2019). SUMO Ylation Promotes Protective Responses to DNA ‐protein Crosslinks. EMBO J. 38, e101496. 10.15252/embj.2019101496 30914427 PMC6463212

[B18] Bradley A. I. Marsh N. M. Borror H. R. Mostoller K. E. Gama A. I. Gardner R. G. (2021). Acute Ethanol Stress Induces Sumoylation of Conserved Chromatin Structural Proteins in *Saccharomyces cerevisiae* . MBoC 32, E20–E11. 10.1091/mbc.E20-11-0715 PMC8351541 33788582

[B19] Bush N. G. Evans-Roberts K. Maxwell A. (2015). DNA Topoisomerases. EcoSal Plus 6. 10.1128/ecosalplus.ESP-0010-2014 PMC11575854 26435256

[B20] Celen A. B. Sahin U. (2020). Sumoylation on its 25th Anniversary: Mechanisms, Pathology, and Emerging Concepts. FEBS J. 287, 3110–3140. 10.1111/febs.15319 32255256

[B21] Chaly N. Brown D. L. (1996). Is DNA Topoisomerase IIβ a Nucleolar Protein? J. Cel. Biochem. 63, 162–173. 10.1002/(sici)1097-4644(19961101)63:2<162::aid-jcb4>3.0.co;2-w 8913868

[B22] Champoux J. J. (2001). DNA Topoisomerases: Structure, Function, and Mechanism. Annu. Rev. Biochem. 70, 369–413. 10.1146/annurev.biochem.70.1.369 11395412

[B23] Chang Y.-C. Oram M. K. Bielinsky A.-K. (2021). SUMO-Targeted Ubiquitin Ligases and Their Functions in Maintaining Genome Stability. Ijms 22, 5391. 10.3390/ijms22105391 34065507 PMC8161396

[B24] Chen M. Rockel T. Steinweger G. Hemmerich P. Risch J. von Mikecz A. (2002). Subcellular Recruitment of Fibrillarin to Nucleoplasmic Proteasomes: Implications for Processing of a Nucleolar Autoantigen. MBoC 13, 3576–3587. 10.1091/mbc.02-05-0083 12388758 PMC129967

[B25] Chen X. L. Silver H. R. Xiong L. Belichenko I. Adegite C. Johnson E. S. (2007). Topoisomerase I-dependent Viability Loss in saccharomyces Cerevisiae Mutants Defective in Both SUMO Conjugation and DNA Repair. Genetics 177, 17–30. 10.1534/genetics.107.074708 17603101 PMC2013680

[B26] Chen S. H. Chan N.-L. Hsieh T.-s. (2013). New Mechanistic and Functional Insights into DNA Topoisomerases. Annu. Rev. Biochem. 82, 139–170. 10.1146/annurev-biochem-061809-100002 23495937

[B27] Christensen M. O. Krokowski R. M. Barthelmes H. U. Hock R. Boege F. Mielke C. (2004). Distinct Effects of Topoisomerase I and RNA Polymerase I Inhibitors Suggest a Dual Mechanism of Nucleolar/nucleoplasmic Partitioning of Topoisomerase I. J. Biol. Chem. 279, 21873–21882. 10.1074/jbc.M400498200 15014084

[B28] Ciechanover A. Stanhill A. (2014). The Complexity of Recognition of Ubiquitinated Substrates by the 26S Proteasome. Biochim. Biophys. Acta (Bba) - Mol. Cel Res. 1843, 86–96. 10.1016/j.bbamcr.2013.07.007 23872423

[B29] Da Costa I. C. Schmidt C. K. (2020). Ubiquitin-like Proteins in the DNA Damage Response: the Next Generation. Essays Biochem. 64, 737–752. 10.1042/EBC20190095 32451552

[B30] D'Arpa P. Beardmore C. Liu L. F. (1990). Involvement of Nucleic Acid Synthesis in Cell Killing Mechanisms of Topoisomerase Poisons. Cancer Res. 50, 6919–6924. 1698546

[B31] Dasgupta T. Ferdous S. Tse-Dinh Y.-C. (2020). Mechanism of Type IA Topoisomerases. Molecules 25, 4769. 10.3390/molecules25204769 PMC7587558 33080770

[B32] Dawlaty M. M. Malureanu L. Jeganathan K. B. Kao E. Sustmann C. Tahk S. (2008). Resolution of Sister Centromeres Requires RanBP2-Mediated SUMOylation of Topoisomerase IIα. Cell 133, 103–115. 10.1016/j.cell.2008.01.045 18394993 PMC2693193

[B33] Debethune L. Kohlhagen G. Grandas A. Pommier Y. (2002). Processing of Nucleopeptides Mimicking the Topoisomerase I-DNA Covalent Complex by Tyrosyl-DNA Phosphodiesterase. Nucleic Acids Res. 30, 1198–1204. 10.1093/nar/30.5.1198 11861912 PMC101246

[B34] Dekker N. H. Rybenkov V. V. Duguet M. Crisona N. J. Cozzarelli N. R. Bensimon D. (2002). The Mechanism of Type IA Topoisomerases. Proc. Natl. Acad. Sci. U.S.A. 99, 12126–12131. 10.1073/pnas.132378799 12167668 PMC129409

[B35] Desai S. D. Liu L. F. Vazquez-Abad D. D'Arpa P. (1997). Ubiquitin-dependent Destruction of Topoisomerase I Is Stimulated by the Antitumor Drug Camptothecin. J. Biol. Chem. 272, 24159–24164. 10.1074/jbc.272.39.24159 9305865

[B36] Desai S. D. Li T. K. Rodriguez-Bauman A. Rubin E. H. Liu L. F. (2001). Ubiquitin/26S Proteasome-Mediated Degradation of Topoisomerase I as a Resistance Mechanism to Camptothecin in Tumor Cells. Cancer Res. 61, 5926–5932. 11479235

[B37] Desai S. D. Zhang H. Rodriguez-Bauman A. Yang J.-M. Wu X. Gounder M. K. (2003). Transcription-dependent Degradation of Topoisomerase I-DNA Covalent Complexes. Mol. Cel Biol 23, 2341–2350. 10.1128/MCB.23.7.2341-2350.2003 PMC150741 12640119

[B38] Dhingra N. Zhao X. (2021). Advances in SUMO-Based Regulation of Homologous Recombination. Curr. Opin. Genet. Dev. 71, 114–119. 10.1016/j.gde.2021.07.007 34333341 PMC8671156

[B39] Díaz-Martínez L. A. Giménez-Abián J. F. Azuma Y. Guacci V. Giménez-Martín G. Lanier L. M. (2006). PIASγ Is Required for Faithful Chromosome Segregation in Human Cells. PLoS One 1, e53. 10.1371/journal.pone.0000053 17183683 PMC1762334

[B40] Dokshin G. A. Davis G. M. Sawle A. D. Eldridge M. D. Nicholls P. K. Gourley T. E. (2020). GCNA Interacts with Spartan and Topoisomerase II to Regulate Genome Stability. Dev. Cel. 52, 53–68.e6. 10.1016/j.devcel.2019.11.006 PMC7227305 31839538

[B41] Edgerton H. Johansson M. Keifenheim D. Mukherjee S. Chacón J. M. Bachant J. (2016). A Noncatalytic Function of the Topoisomerase II CTD in Aurora B Recruitment to Inner Centromeres during Mitosis. J. Cel. Biol. 213, 651–664. 10.1083/jcb.201511080 PMC4915189 27325791

[B42] Enenkel C. (2014). Proteasome Dynamics. Biochim. Biophys. Acta (Bba) - Mol. Cel Res. 1843, 39–46. 10.1016/j.bbamcr.2013.03.023 23545412

[B43] Fielden J. Wiseman K. Torrecilla I. Li S. Hume S. Chiang S.-C. (2020). TEX264 Coordinates P97- and SPRTN-Mediated Resolution of Topoisomerase 1-DNA Adducts. Nat. Commun. 11, 1274. 10.1038/s41467-020-15000-w 32152270 PMC7062751

[B44] Flotho A. Melchior F. (2013). Sumoylation: a Regulatory Protein Modification in Health and Disease. Annu. Rev. Biochem. 82, 357–385. 10.1146/annurev-biochem-061909-093311 23746258

[B45] Galanty Y. Belotserkovskaya R. Coates J. Polo S. Miller K. M. Jackson S. P. (2009). Mammalian SUMO E3-Ligases PIAS1 and PIAS4 Promote Responses to DNA Double-Strand Breaks. Nature 462, 935–939. 10.1038/nature08657 20016603 PMC2904806

[B46] Galanty Y. Belotserkovskaya R. Coates J. Jackson S. P. (2012). RNF4, a SUMO-Targeted Ubiquitin E3 Ligase, Promotes DNA Double-Strand Break Repair. Genes Dev. 26, 1179–1195. 10.1101/gad.188284.112 22661229 PMC3371407

[B47] Gao R. Schellenberg M. J. Huang S.-y. N. Abdelmalak M. Marchand C. Nitiss K. C. (2014). Proteolytic Degradation of Topoisomerase II (Top2) Enables the Processing of Top2·DNA and Top2·RNA Covalent Complexes by Tyrosyl-DNA-Phosphodiesterase 2 (TDP2). J. Biol. Chem. 289, 17960–17969. 10.1074/jbc.M114.565374 24808172 PMC4140274

[B48] Geiss-Friedlander R. Melchior F. (2007). Concepts in Sumoylation: a Decade on. Nat. Rev. Mol. Cel. Biol. 8, 947–956. 10.1038/nrm2293 18000527

[B49] Gonzalez R. E. Lim C.-U. Cole K. Bianchini C. H. Schools G. P. Davis B. E. (2011). Effects of Conditional Depletion of Topoisomerase II on Cell Cycle Progression in Mammalian Cells. Cell Cycle 10, 3505–3514. 10.4161/cc.10.20.17778 22067657 PMC3356834

[B50] Guturi K. K. N. Bohgaki M. Bohgaki T. Srikumar T. Ng D. Kumareswaran R. (2016). RNF168 and USP10 Regulate Topoisomerase IIα Function via Opposing Effects on its Ubiquitylation. Nat. Commun. 7, 12638. 10.1038/ncomms12638 27558965 PMC5007378

[B51] Hammer E. Heilbronn R. Weger S. (2007). The E3 Ligase Topors Induces the Accumulation of Polysumoylated Forms of DNA Topoisomerase I *In Vitro* and *In Vivo* . FEBS Lett. 581, 5418–5424. 10.1016/j.febslet.2007.10.040 17976381

[B52] Hauk G. Berger J. M. (2016). The Role of ATP-dependent Machines in Regulating Genome Topology. Curr. Opin. Struct. Biol. 36, 85–96. 10.1016/j.sbi.2016.01.006 26827284 PMC4785063

[B53] Heideker J. Prudden J. Perry J. J. P. Tainer J. A. Boddy M. N. (2011). SUMO-targeted Ubiquitin Ligase, Rad60, and Nse2 SUMO Ligase Suppress Spontaneous Top1-Mediated DNA Damage and Genome Instability. Plos Genet. 7, e1001320. 10.1371/journal.pgen.1001320 21408210 PMC3048374

[B54] Hendriks I. A. D'Souza R. C. J. Yang B. Verlaan-de Vries M. Mann M. Vertegaal A. C. O. (2014). Uncovering Global SUMOylation Signaling Networks in a Site-specific Manner. Nat. Struct. Mol. Biol. 21, 927–936. 10.1038/nsmb.2890 25218447 PMC4259010

[B55] Hochstrasser M. (2001). SP-RING for SUMO: New Functions Bloom for a Ubiquitin-like Protein. Cell 107, 5–8. 10.1016/s0092-8674(01)00519-0 11595179

[B56] Horie K. Tomida A. Sugimoto Y. Yasugi T. Yoshikawa H. Taketani Y. (2002). SUMO-1 Conjugation to Intact DNA Topoisomerase I Amplifies Cleavable Complex Formation Induced by Camptothecin. Oncogene 21, 7913–7922. 10.1038/sj.onc.1205917 12439742

[B57] Horigome C. Bustard D. E. Marcomini I. Delgoshaie N. Tsai-Pflugfelder M. Cobb J. A. (2016). PolySUMOylation by Siz2 and Mms21 Triggers Relocation of DNA Breaks to Nuclear Pores through the Slx5/Slx8 STUbL. Genes Dev. 30, 931–945. 10.1101/gad.277665.116 27056668 PMC4840299

[B58] Hornbeck P. V. Zhang B. Murray B. Kornhauser J. M. Latham V. Skrzypek E. (2015). PhosphoSitePlus, 2014: Mutations, PTMs and Recalibrations. Nucleic Acids Res. 43, D512–D520. 10.1093/nar/gku1267 25514926 PMC4383998

[B59] Hudson J. J. R. Chiang S.-C. Wells O. S. Rookyard C. El-Khamisy S. F. (2012). SUMO Modification of the Neuroprotective Protein TDP1 Facilitates Chromosomal Single-Strand Break Repair. Nat. Commun. 3, 733. 10.1038/ncomms1739 22415824 PMC3316882

[B60] Interthal H. Chen H. J. Champoux J. J. (2005). Human Tdp1 Cleaves a Broad Spectrum of Substrates, Including Phosphoamide Linkages. J. Biol. Chem. 280, 36518–36528. 10.1074/jbc.M508898200 16141202 PMC1351008

[B61] Isik S. Sano K. Tsutsui K. Seki M. Enomoto T. Saitoh H. (2003). The SUMO Pathway Is Required for Selective Degradation of DNA Topoisomerase IIβ Induced by a Catalytic Inhibitor ICRF-193(1). FEBS Lett. 546, 374–378. 10.1016/s0014-5793(03)00637-9 12832072

[B62] Jacquiau H. R. van Waardenburg R. C. A. M. Reid R. J. D. Woo M. H. Guo H. Johnson E. S. (2005). Defects in SUMO (Small Ubiquitin-Related Modifier) Conjugation and Deconjugation Alter Cell Sensitivity to DNA Topoisomerase I-Induced DNA Damage. J. Biol. Chem. 280, 23566–23575. 10.1074/jbc.M500947200 15817450

[B63] Jalal D. Chalissery J. Hassan A. H. (2017). Genome Maintenance in Saccharomyces Cerevisiae: the Role of SUMO and SUMO-Targeted Ubiquitin Ligases. Nucleic Acids Res. 45, gkw1369–2261. 10.1093/nar/gkw1369 PMC5389695 28115630

[B64] Kanagasabai R. Liu S. Salama S. Yamasaki E. F. Zhang L. Greenchurch K. B. (2009). Ubiquitin-family Modifications of Topoisomerase I in Camptothecin-Treated Human Breast Cancer Cells. Biochemistry 48, 3176–3185. 10.1021/bi802179t 19236054 PMC2693397

[B65] Kojima Y. Machida Y. Palani S. Caulfield T. R. Radisky E. S. Kaufmann S. H. (2020). FAM111A Protects Replication forks from Protein Obstacles via its Trypsin-like Domain. Nat. Commun. 11, 1318. 10.1038/s41467-020-15170-7 32165630 PMC7067828

[B66] Krastev D. B. Li S. Sun Y. Wicks A. J. Hoslett G. Weekes D. (2022). The Ubiquitin-dependent ATPase P97 Removes Cytotoxic Trapped PARP1 from Chromatin. Nat. Cel. Biol. 24, 62–73. 10.1038/s41556-021-00807-6 PMC8760077 35013556

[B67] Kravtsova-Ivantsiv Y. Ciechanover A. (2012). Non-canonical Ubiquitin-Based Signals for Proteasomal Degradation. J. Cel. Sci. 125, 539–548. 10.1242/jcs.093567 22389393

[B141] Kramarz K. Schirmeisen K. Boucherit V. Saada A. A. Lovo C. Palancade B. (2020). The Nuclear Pore Primes Recombination-Dependent DNA Synthesis at Arrested Forks by Promoting SUMO Removal. Nat. Commun. 11 (1), 5643. 10.1038/s41467-020-19516-z 33159083 PMC7648084

[B68] Kreuzer K. N. Cozzarelli N. R. (1980). Formation and Resolution of DNA Catenanes by DNA Gyrase. Cell 20, 245–254. 10.1016/0092-8674(80)90252-4 6248235

[B69] Kumar R. González-Prieto R. Xiao Z. Verlaan-de Vries M. Vertegaal A. C. O. (2017). The STUbL RNF4 Regulates Protein Group SUMOylation by Targeting the SUMO Conjugation Machinery. Nat. Commun. 8, 1809. 10.1038/s41467-017-01900-x 29180619 PMC5703878

[B70] Kunz K. Piller T. Müller S. (2018). SUMO-specific Proteases and Isopeptidases of the SENP Family at a Glance. J. Cel. Sci. 131, jcs211904. 10.1242/jcs.211904 29559551

[B71] Kuo C.-Y. Li X. Stark J. M. Shih H.-M. Ann D. K. (2016). RNF4 Regulates DNA Double-Strand Break Repair in a Cell Cycle-dependent Manner. Cell Cycle 15, 787–798. 10.1080/15384101.2016.1138184 26766492 PMC4845925

[B72] Lee M.-T. Bachant J. (2009). SUMO Modification of DNA Topoisomerase II: Trying to Get a CENse of it All. DNA Repair 8, 557–568. 10.1016/j.dnarep.2009.01.004 19230795 PMC2688443

[B73] Levin N. A. Bjornsti M. A. Fink G. R. (1993). A Novel Mutation in DNA Topoisomerase I of Yeast Causes DNA Damage and RAD9-dependent Cell Cycle Arrest. Genetics 133, 799–814. 10.1093/genetics/133.4.799 8385050 PMC1205401

[B74] Li M. Pokharel S. Wang J.-T. Xu X. Liu Y. (2015). RECQ5-dependent SUMOylation of DNA Topoisomerase I Prevents Transcription-Associated Genome Instability. Nat. Commun. 6, 6720. 10.1038/ncomms7720 25851487 PMC7553879

[B75] Lin C.-P. Ban Y. Lyu Y. L. Desai S. D. Liu L. F. (2008). A Ubiquitin-Proteasome Pathway for the Repair of Topoisomerase I-DNA Covalent Complexes. J. Biol. Chem. 283, 21074–21083. 10.1074/jbc.M803493200 18515798 PMC2475699

[B76] Lin C.-P. Ban Y. Lyu Y. L. Liu L. F. (2009). Proteasome-dependent Processing of Topoisomerase I-DNA Adducts into DNA Double Strand Breaks at Arrested Replication forks. J. Biol. Chem. 284, 28084–28092. 10.1074/jbc.M109.030601 19666469 PMC2788859

[B77] Linka R. M. Porter A. C. G. Volkov A. Mielke C. Boege F. Christensen M. O. (2007). C-Terminal Regions of Topoisomerase II and II Determine Isoform-specific Functioning of the Enzymes *In Vivo* . Nucleic Acids Res. 35, 3810–3822. 10.1093/nar/gkm102 17526531 PMC1920234

[B78] Liu J. C. Y. Kühbacher U. Larsen N. B. Borgermann N. Garvanska D. H. Hendriks I. A. (2021). Mechanism and Function of DNA Replication‐independent DNA‐protein Crosslink Repair via the SUMO‐RNF4 Pathway. EMBO J. 40, e107413. 10.15252/embj.2020107413 34346517 PMC8441304

[B79] Maeshima K. Laemmli U. K. (2003). A Two-step Scaffolding Model for Mitotic Chromosome Assembly. Dev. Cel. 4, 467–480. 10.1016/s1534-5807(03)00092-3 12689587

[B80] Mao Y. Desai S. D. Liu L. F. (2000a). SUMO-1 Conjugation to Human DNA Topoisomerase II Isozymes. J. Biol. Chem. 275, 26066–26073. 10.1074/jbc.M001831200 10862613

[B81] Mao Y. Sun M. Desai S. D. Liu L. F. (2000b). SUMO-1 Conjugation to Topoisomerase I: A Possible Repair Response to Topoisomerase-Mediated DNA Damage. Proc. Natl. Acad. Sci. U.S.A. 97, 4046–4051. 10.1073/pnas.080536597 10759568 PMC18143

[B82] Mao Y. Desai S. D. Ting C.-Y. Hwang J. Liu L. F. (2001). 26 S Proteasome-Mediated Degradation of Topoisomerase II Cleavable Complexes. J. Biol. Chem. 276, 40652–40658. 10.1074/jbc.M104009200 11546768

[B83] Matic I. van Hagen M. Schimmel J. Macek B. Ogg S. C. Tatham M. H. (2008). *In Vivo* identification of Human Small Ubiquitin-like Modifier Polymerization Sites by High Accuracy Mass Spectrometry and an *In Vitro* to *In Vivo* Strategy. Mol. Cell Proteomics 7, 132–144. 10.1074/mcp.M700173-MCP200 17938407 PMC3840926

[B84] Matlock M. K. Holehouse A. S. Naegle K. M. (2015). ProteomeScout: a Repository and Analysis Resource for post-translational Modifications and Proteins. Nucleic Acids Res. 43, D521–D530. 10.1093/nar/gku1154 25414335 PMC4383955

[B85] McClendon A. K. Gentry A. C. Dickey J. S. Brinch M. Bendsen S. Andersen A. H. (2008). Bimodal Recognition of DNA Geometry by Human Topoisomerase IIα: Preferential Relaxation of Positively Supercoiled DNA Requires Elements in the C-Terminal Domain. Biochemistry 47, 13169–13178. 10.1021/bi800453h 19053267 PMC2629653

[B86] McKie S. J. Neuman K. C. Maxwell A. (2021). DNA Topoisomerases: Advances in Understanding of Cellular Roles and Multi‐protein Complexes via Structure‐Function Analysis. Bioessays 43, 2000286. 10.1002/bies.202000286 PMC7614492 33480441

[B87] Miteva M. Keusekotten K. Hofmann K. Praefcke G. J. K. Dohmen R. J. (2010). Sumoylation as a Signal for Polyubiquitylation and Proteasomal Degradation. Subcell Biochem. 54, 195–214. 10.1007/978-1-4419-6676-6_16 21222284

[B88] Mo Y.-Y. Yu Y. Shen Z. Beck W. T. (2002). Nucleolar Delocalization of Human Topoisomerase I in Response to Topotecan Correlates with Sumoylation of the Protein. J. Biol. Chem. 277, 2958–2964. 10.1074/jbc.M108263200 11709553

[B89] Mukhopadhyay D. Dasso M. (2007). Modification in Reverse: the SUMO Proteases. Trends Biochem. Sci. 32, 286–295. 10.1016/j.tibs.2007.05.002 17499995

[B90] Mukhopadhyay D. Dasso M. (2017). The SUMO Pathway in Mitosis. Adv. Exp. Med. Biol. 963, 171–184. 10.1007/978-3-319-50044-7_10 28197912 PMC11166265

[B91] Murai J. Huang S.-y. N. Das B. B. Dexheimer T. S. Takeda S. Pommier Y. (2012). Tyrosyl-DNA Phosphodiesterase 1 (TDP1) Repairs DNA Damage Induced by Topoisomerases I and II and Base Alkylation in Vertebrate Cells. J. Biol. Chem. 287, 12848–12857. 10.1074/jbc.M111.333963 22375014 PMC3339927

[B92] Nayak A. Müller S. (2014). SUMO-specific Proteases/isopeptidases: SENPs and beyond. Genome Biol. 15, 422. 10.1186/s13059-014-0422-2 25315341 PMC4281951

[B93] Nie M. Aslanian A. Prudden J. Heideker J. Vashisht A. A. Wohlschlegel J. A. (2012). Dual Recruitment of Cdc48 (P97)-Ufd1-Npl4 Ubiquitin-Selective Segregase by Small Ubiquitin-like Modifier Protein (SUMO) and Ubiquitin in SUMO-Targeted Ubiquitin Ligase-Mediated Genome Stability Functions. J. Biol. Chem. 287, 29610–29619. 10.1074/jbc.M112.379768 22730331 PMC3436128

[B94] Nitiss K. C. Malik M. He X. White S. W. Nitiss J. L. (2006). Tyrosyl-DNA Phosphodiesterase (Tdp1) Participates in the Repair of Top2-Mediated DNA Damage. Proc. Natl. Acad. Sci. U.S.A. 103, 8953–8958. 10.1073/pnas.0603455103 16751265 PMC1482547

[B95] Nitiss K. C. Nitiss J. L. Hanakahi L. A. (2019). DNA Damage by an Essential Enzyme: A Delicate Balance Act on the Tightrope. DNA Repair 82, 102639. 10.1016/j.dnarep.2019.102639 31437813

[B96] Nitiss J. L. (2009a). DNA Topoisomerase II and its Growing Repertoire of Biological Functions. Nat. Rev. Cancer 9, 327–337. 10.1038/nrc2608 19377505 PMC2730144

[B97] Nitiss J. L. (2009b). Targeting DNA Topoisomerase II in Cancer Chemotherapy. Nat. Rev. Cancer 9, 338–350. 10.1038/nrc2607 19377506 PMC2748742

[B98] Pandey N. Keifenheim D. Yoshida M. M. Hassebroek V. A. Soroka C. Azuma Y. (2020). Topoisomerase II SUMOylation Activates a Metaphase Checkpoint via Haspin and Aurora B Kinases. J. Cel. Biol. 219, e201807189. 10.1083/jcb.201807189 PMC7039214 31712254

[B99] Paulson J. R. Hudson D. F. Cisneros-Soberanis F. Earnshaw W. C. (2021). Mitotic Chromosomes. Semin. Cel. Dev. Biol. 117, 7–29. 10.1016/j.semcdb.2021.03.014 PMC8406421 33836947

[B100] Pichler A. Melchior F. (2002). Ubiquitin-related Modifier SUMO1 and Nucleocytoplasmic Transport. Traffic 3, 381–387. 10.1034/j.1600-0854.2002.30601.x 12010456

[B101] Pichler A. Knipscheer P. Saitoh H. Sixma T. K. Melchior F. (2004). The RanBP2 SUMO E3 Ligase Is Neither HECT- Nor RING-type. Nat. Struct. Mol. Biol. 11, 984–991. 10.1038/nsmb834 15378033

[B102] Pichler A. Fatouros C. Lee H. Eisenhardt N. (2017). SUMO Conjugation - a Mechanistic View. Biomol. Concepts 8, 13–36. 10.1515/bmc-2016-0030 28284030

[B140] Pommier Y. Nussenzweig A. Takeda S. Austin C. (2022). Human Topoisomerases and their Roles in Genome Stability and Organization. Nat. Rev. Mol. Cell Biol. 28, 1–21. 10.1038/s41580-022-00452-3 PMC8883456 35228717

[B103] Pommier Y. Sun Y. Huang S.-y. N. Nitiss J. L. (2016). Roles of Eukaryotic Topoisomerases in Transcription, Replication and Genomic Stability. Nat. Rev. Mol. Cel Biol. 17, 703–721. 10.1038/nrm.2016.111 PMC9248348 27649880

[B104] Pommier Y. (2006). Topoisomerase I Inhibitors: Camptothecins and beyond. Nat. Rev. Cancer 6, 789–802. 10.1038/nrc1977 16990856

[B105] Pouliot J. J. Yao K. C. Robertson C. A. Nash H. A. (1999). Yeast Gene for a Tyr-DNA Phosphodiesterase that Repairs Topoisomerase I Complexes. Science 286, 552–555. 10.1126/science.286.5439.552 10521354

[B106] Psakhye I. Castellucci F. Branzei D. (2019). SUMO-Chain-Regulated Proteasomal Degradation Timing Exemplified in DNA Replication Initiation. Mol. Cel 76, 632–645.e6. 10.1016/j.molcel.2019.08.003 PMC6891891 31519521

[B107] Rallabhandi P. Hashimoto K. Mo Y.-Y. Beck W. T. Moitra P. K. D'Arpa P. (2002). Sumoylation of Topoisomerase I Is Involved in its Partitioning between Nucleoli and Nucleoplasm and its Clearing from Nucleoli in Response to Camptothecin. J. Biol. Chem. 277, 40020–40026. 10.1074/jbc.M200388200 12149243

[B108] Riccio A. A. Schellenberg M. J. Williams R. S. (2020). Molecular Mechanisms of Topoisomerase 2 DNA-Protein Crosslink Resolution. Cell. Mol. Life Sci. 77, 81–91. 10.1007/s00018-019-03367-z 31728578 PMC6960353

[B109] Ruggiano A. Vaz B. Kilgas S. Popović M. Rodriguez-Berriguete G. Singh A. N. (2021). The Protease SPRTN and SUMOylation Coordinate DNA-Protein Crosslink Repair to Prevent Genome Instability. Cel Rep. 37, 110080. 10.1016/j.celrep.2021.110080 PMC8674535 34879279

[B110] Rytinki M. M. Kaikkonen S. Pehkonen P. Jääskeläinen T. Palvimo J. J. (2009). PIAS Proteins: Pleiotropic Interactors Associated with SUMO. Cel. Mol. Life Sci. 66, 3029–3041. 10.1007/s00018-009-0061-z PMC11115825 19526197

[B111] Ryu H. Furuta M. Kirkpatrick D. Gygi S. P. Azuma Y. (2010). PIASy-Dependent SUMOylation Regulates DNA Topoisomerase IIα Activity. J. Cel Biol 191, 783–794. 10.1083/jcb.201004033 PMC2983052 21079245

[B112] Ryu H. Yoshida M. M. Sridharan V. Kumagai A. Dunphy W. G. Dasso M. (2015). SUMOylation of the C-Terminal Domain of DNA Topoisomerase IIα Regulates the Centromeric Localization of Claspin. Cell Cycle 14, 2777–2784. 10.1080/15384101.2015.1066537 26131587 PMC4614044

[B113] Schellenberg M. J. Lieberman J. A. Herrero-Ruiz A. Butler L. R. Williams J. G. Muñoz-Cabello A. M. (2017). ZATT (ZNF451)-Mediated Resolution of Topoisomerase 2 DNA-Protein Cross-Links. Science 357, 1412–1416. 10.1126/science.aam6468 28912134 PMC5623066

[B114] Seol Y. Neuman K. C. (2016). The Dynamic Interplay between DNA Topoisomerases and DNA Topology. Biophys. Rev. 8, 221–231. 10.1007/s12551-016-0206-x 27942270 PMC5145260

[B115] Serbyn N. Noireterre A. Bagdiul I. Plank M. Michel A. H. Loewith R. (2020). The Aspartic Protease Ddi1 Contributes to DNA-Protein Crosslink Repair in Yeast. Mol. Cel. 77, 1066–1079.e9. 10.1016/j.molcel.2019.12.007 31902667

[B116] Serbyn N. Bagdiul I. Noireterre A. Michel A. H. Suhandynata R. T. Zhou H. (2021). SUMO Orchestrates Multiple Alternative DNA-Protein Crosslink Repair Pathways. Cel Rep. 37, 110034. 10.1016/j.celrep.2021.110034 PMC10042627 34818558

[B117] Shin E. J. Shin H. M. Nam E. Kim W. S. Kim J. H. Oh B. H. (2012). DeSUMOylating Isopeptidase: a Second Class of SUMO Protease. EMBO Rep. 13, 339–346. 10.1038/embor.2012.3 22370726 PMC3321169

[B118] Shintomi K. Hirano T. (2018). Reconstitution of Mitotic Chromatids *In Vitro* . Curr. Protoc. Cel Biol. 79, e48. 10.1002/cpcb.48 29924489

[B119] Stantial N. Rogojina A. Gilbertson M. Sun Y. Miles H. Shaltz S. (2020). Trapped Topoisomerase II Initiates Formation of De Novo Duplications via the Nonhomologous End-Joining Pathway in Yeast. Proc. Natl. Acad. Sci. U.S.A. 117, 26876–26884. 10.1073/pnas.2008721117 33046655 PMC7604471

[B120] Steinacher R. Osman F. Lorenz A. Bryer C. Whitby M. C. (2013). Slx8 Removes Pli1-dependent Protein-SUMO Conjugates Including SUMOylated Topoisomerase I to Promote Genome Stability. PLoS One 8, e71960. 10.1371/journal.pone.0071960 23936535 PMC3735562

[B121] Sun Y. Miller Jenkins L. M. Su Y. P. Nitiss K. C. Nitiss J. L. Pommier Y. (2020a). A Conserved SUMO Pathway Repairs Topoisomerase DNA-Protein Cross-Links by Engaging Ubiquitin-Mediated Proteasomal Degradation. Sci. Adv. 6, eaba6290. 10.1126/sciadv.aba6290 33188014 PMC7673754

[B122] Sun Y. Saha L. K. Saha S. Jo U. Pommier Y. (2020b). Debulking of Topoisomerase DNA-Protein Crosslinks (TOP-DPC) by the Proteasome, Non-proteasomal and Non-proteolytic Pathways. DNA Repair 94, 102926. 10.1016/j.dnarep.2020.102926 32674013 PMC9210512

[B123] Sundin O. Varshavsky A. (1981). Arrest of Segregation Leads to Accumulation of Highly Intertwined Catenated Dimers: Dissection of the Final Stages of SV40 DNA Replication. Cell 25, 659–669. 10.1016/0092-8674(81)90173-2 6269752

[B124] Takahashi Y. Yong-Gonzalez V. Kikuchi Y. Strunnikov A. (2006). SIZ1/SIZ2 Control of Chromosome Transmission Fidelity Is Mediated by the Sumoylation of Topoisomerase II. Genetics 172, 783–794. 10.1534/genetics.105.047167 16204216 PMC1456244

[B125] Takahashi D. T. Gadelle D. Agama K. Kiselev E. Zhang H. Yab E. (2022). Topoisomerase I (TOP1) Dynamics: Conformational Transition from Open to Closed States. Nat. Commun. 13, 59. 10.1038/s41467-021-27686-7 35013228 PMC8748870

[B126] Tian T. Bu M. Chen X. Ding L. Yang Y. Han J. (2021). The ZATT-TOP2A-PICH Axis Drives Extensive Replication Fork Reversal to Promote Genome Stability. Mol. Cel. 81, 198–211.e6. 10.1016/j.molcel.2020.11.007 33296677

[B127] Uzunova K. Göttsche K. Miteva M. Weisshaar S. R. Glanemann C. Schnellhardt M. (2007). Ubiquitin-dependent Proteolytic Control of SUMO Conjugates. J. Biol. Chem. 282, 34167–34175. 10.1074/jbc.M706505200 17728242

[B128] van den Boom J. Meyer H. (2018). VCP/p97-Mediated Unfolding as a Principle in Protein Homeostasis and Signaling. Mol. Cel. 69, 182–194. 10.1016/j.molcel.2017.10.028 29153394

[B129] Vos S. M. Tretter E. M. Schmidt B. H. Berger J. M. (2011). All Tangled up: How Cells Direct, Manage and Exploit Topoisomerase Function. Nat. Rev. Mol. Cel. Biol. 12, 827–841. 10.1038/nrm3228 PMC4351964 22108601

[B130] Vyas R. Kumar R. Clermont F. Helfricht A. Kalev P. Sotiropoulou P. (2013). RNF4 Is Required for DNA Double-Strand Break Repair *In Vivo* . Cell Death Differ. 20, 490–502. 10.1038/cdd.2012.145 23197296 PMC3569989

[B131] Xiao H. Mao Y. Desai S. D. Zhou N. Ting C.-Y. Hwang J. (2003). The Topoisomerase IIβ Circular Clamp Arrests Transcription and Signals a 26S Proteasome Pathway. Proc. Natl. Acad. Sci. U.S.A. 100, 3239–3244. 10.1073/pnas.0736401100 12629207 PMC152276

[B132] Yang S. W. Burgin A. B. Jr. Huizenga B. N. Robertson C. A. Yao K. C. Nash H. A. (1996). A Eukaryotic Enzyme that Can Disjoin Dead-End Covalent Complexes between DNA and Type I Topoisomerases. Proc. Natl. Acad. Sci. 93, 11534–11539. 10.1073/pnas.93.21.11534 8876170 PMC38092

[B133] Yang M. Hsu C.-T. Ting C.-Y. Liu L. F. Hwang J. (2006). Assembly of a Polymeric Chain of SUMO1 on Human Topoisomerase I *In Vitro* . J. Biol. Chem. 281, 8264–8274. 10.1074/jbc.M510364200 16428803

[B134] Yin Y. Seifert A. Chua J. S. Maure J.-F. Golebiowski F. Hay R. T. (2012). SUMO-targeted Ubiquitin E3 Ligase RNF4 Is Required for the Response of Human Cells to DNA Damage. Genes Dev. 26, 1196–1208. 10.1101/gad.189274.112 22661230 PMC3371408

[B135] Yoshida M. M. Azuma Y. (2016). Mechanisms behind Topoisomerase II SUMOylation in Chromosome Segregation. Cell Cycle 15, 3151–3152. 10.1080/15384101.2016.1216928 27484981 PMC5176152

[B136] Yoshida M. M. Ting L. Gygi S. P. Azuma Y. (2016). SUMOylation of DNA Topoisomerase IIα Regulates Histone H3 Kinase Haspin and H3 Phosphorylation in Mitosis. J. Cel. Biol. 213, 665–678. 10.1083/jcb.201511079 PMC4915188 27325792

[B137] Zagnoli-Vieira G. Caldecott K. W. (2020). Untangling Trapped Topoisomerases with Tyrosyl-DNA Phosphodiesterases. DNA Repair 94, 102900. 10.1016/j.dnarep.2020.102900 32653827

[B138] Zhang H. Barceló J. M. Lee B. Kohlhagen G. Zimonjic D. B. Popescu N. C. (2001). Human Mitochondrial Topoisomerase I. Proc. Natl. Acad. Sci. U.S.A. 98, 10608–10613. 10.1073/pnas.191321998 11526219 PMC58513

[B139] Zhang L. Wang S. Yin S. Hong S. Kim K. P. Kleckner N. (2014). Topoisomerase II Mediates Meiotic Crossover Interference. Nature 511, 551–556. 10.1038/nature13442 25043020 PMC4128387

